# Measuring hospital‐specific disparities by dual eligibility and race to reduce health inequities

**DOI:** 10.1111/1475-6773.13108

**Published:** 2019-01-21

**Authors:** Anouk Lloren, Shuling Liu, Jeph Herrin, Zhenqiu Lin, Guohai Zhou, Yongfei Wang, Meng Kuang, Sheng Zhou, Thalia Farietta, Kerry McCole, Sana Charania, Karen Dorsey Sheares, Susannah Bernheim

**Affiliations:** ^1^ Center for Outcomes Research and Evaluation (CORE) Yale New Haven Hospital New Haven Connecticut; ^2^ Section of Cardiovascular Medicine Center for Outcomes Research and Evaluation (CORE) Yale New Haven Hospital New Haven Connecticut; ^3^ Section of Cardiovascular Medicine Center for Outcomes Research and Evaluation (CORE) Yale New Haven Hospital Yale University School of Medicine New Haven Connecticut; ^4^ Section of Cardiovascular Medicine Department of Medicine Center for Outcomes Research and Evaluation (CORE) Yale Univerity New Haven Connecticut; ^5^ Center for Outcomes Research and Evaluation Yale New Haven Hospital New Haven Connecticut; ^6^ Section of Pediatrics Department of Pediatrics Center for Outcomes Research and Evaluation Yale‐New Haven Hospital New Haven Connecticut; ^7^ Section of General Internal Medicine Department of Internal Medicine Center for Outcomes Research and Evaluation Yale New Haven Hospital New Haven Connecticut

**Keywords:** disparities, dual eligibility, quality measurement, race

## Abstract

**Objective:**

To propose and evaluate a metric for quantifying hospital‐specific disparities in health outcomes that can be used by patients and hospitals.

**Data Sources/Study Setting:**

Inpatient admissions for Medicare patients with acute myocardial infarction, heart failure, or pneumonia to all non‐federal, short‐term, acute care hospitals during 2012‐2015.

**Study Design:**

Building on the current Centers for Medicare and Medicaid Services methodology for calculating risk‐standardized readmission rates, we developed models that include a hospital‐specific random coefficient for either patient dual eligibility status or African American race. These coefficients quantify the difference in risk‐standardized outcomes by dual eligibility and race at a given hospital after accounting for the hospital's patient case mix and proportion of dual eligible or African American patients. We demonstrate this approach and report variation and performance in hospital‐specific disparities.

**Principal Findings:**

Dual eligibility and African American race were associated with higher readmission rates within hospitals for all three conditions. However, this disparity effect varied substantially across hospitals.

**Conclusion:**

Our models isolate a hospital‐specific disparity effect and demonstrate variation in quality of care for different groups of patients across conditions and hospitals. Illuminating within‐hospital disparities can incentivize hospitals to reduce inequities in health care quality.

## 1 INTRODUCTION

Over the last decade, the Centers for Medicare and Medicaid Services (CMS) has promoted the use of quality measures in accountability programs with the goal of improving patient health care and well‐being. Under the Affordable Care Act, CMS extended these efforts by establishing pay‐for‐performance programs, which tie payment to the relative performance of hospitals on quality measures. These accountability programs have contributed to improving patient care.^1^, ^2^, ^3^ However, current quality improvement efforts generally do not address improving quality of care specifically for patients with social risk factors, such as low‐income individuals or people of color, even though these groups experience worse health care quality.^4^, ^5^, ^6^

In this context, and with the goal of promoting health care equity, multiple stakeholders have proposed to highlight disparities in outcome measures by social risk factors.^7^, ^8^, ^9^, ^10^ Despite these recommendations and persisting disparities in health outcomes,^11^, ^12^, ^13^, ^14^, ^15^ few quality measures or methods have emerged for highlighting health care disparities, and none are in widespread use.

In order to address this gap in reporting disparities, this study proposes and evaluates a metric to quantify hospital‐specific disparities in health outcomes. Such a metric could be used to target quality improvement efforts to reduce inequalities in health care. Importantly, our proposed approach recognizes that social risk factors can affect outcomes through two mechanisms: between‐hospital effects, where patients with certain risk factors may be more likely to be treated at hospitals with lower overall health care quality for all patients, and within‐hospital effects, where patients with social risk factors may have different outcomes than patients without social risk factors at the same hospital. Research has documented both types of disparity effects.^10^, ^15^, ^16^, ^17^ While we recognize that both are critical to understand, our approach separates the two and isolates the within‐hospital disparity, or hospital‐specific disparity effect, related to social risk factors. By focusing on a hospital‐specific assessment of disparities, this measurement strategy allows hospitals to assess the gaps in care and outcomes among patient groups cared for within their institution and specifically targets a tangible component of health care disparities that hospitals can directly influence.

Our approach also recognizes the multiple ways in which social risk factors can affect outcomes for patients within a hospital. First, patients with social risk factors may have different degrees of clinical illness and comorbid disease. It is important to account for these differences in order to illuminate the hospital‐specific disparity that could be attributed to hospital quality. Another mechanism is differential care, where hospitals systematically provide different treatment to different patients, such as differential rates of procedure,^18^, ^19^, ^20^ or fail to provide adequately differentiated care, such as language interpreters, leading to worse outcomes for patients with social risk factors. Such differences in the quality of care may reflect hospitals’ response to patients’ different ability to pay, implicit or explicit bias toward patients of certain disadvantaged groups, or different assumptions about certain patients’ ability to care for themselves. Finally, social risk factors may influence outcomes more directly; for example, a social risk factor such as “living alone” may increase the risk of being readmitted after discharge regardless of care and comorbidities.^21^

While recognizing these distinctions are important for addressing and mitigating disparities in health outcomes, our approach accounts for differences in clinical status and assumes that other mechanisms can and should be mitigated to some extent by hospitals and, therefore, should be captured by disparity measurement. This assumption is informed by existing data which show that hospitals that care for very high proportions of patients with social risk factors can perform similarly well on hospital quality measures,^4^, ^22^ along with the recognition that hospitals can address the underlying causes independent of the specific mechanisms.^8^, ^23^, ^24^, ^25^, ^26^ Thus, our approach assumes that each hospital has a latent disparity effect—that is, for each hospital a social risk factor will have more or less of an effect on the outcome depending on the degree to which the hospital mitigates some or all of these mechanisms. Importantly, our approach allows us to test this assumption, in that we will derive an estimate of the variation across hospitals of this assumed latent disparity effect.

In this paper, we define a new metric to illuminate within‐hospital differences in outcomes for different patient groups. This metric is an extension of existing quality outcome measures and can be implemented using the same data and cohorts, which will facilitate widespread use. We then apply this approach to three quality measures currently reported through CMS's *Hospital Compare* website: readmission within 30 days after discharge for acute myocardial infarction (AMI), heart failure, and pneumonia. For each of these three measures, we estimate within‐hospital disparities for two social risk factors, dual eligibility for Medicare and Medicaid (“dual eligibility”) and race. Dual eligibility is a marker for poverty and a known predictor of poor health outcomes.^10^ African American patients have also been consistently shown to have differential outcomes compared to White patients.^27^ In demonstrating and reporting the estimated within‐hospital difference in outcomes for these measures, we hope to provide a tool that can be used by consumers and hospitals to identify how well each hospital mitigates social risk among their patients.

## 2 METHODS

### 2.1 Data sources

This study used Medicare administrative claims data for hospitalizations from July 1, 2012 to June 30, 2015. The cohort and patients’ clinical risk factors were identified using the inpatient and outpatient Standard Analytic Files. We linked these data to the Master Beneficiary Summary File (MBSF) to determine a patient's Medicaid and Medicare dual eligibility status. The Medicare Enrollment Database (EDB) provided information on a patient's race.

### 2.2 Study population

We focused on the AMI, heart failure, and pneumonia readmission measure cohorts, which include inpatient admissions to all non‐federal, short‐term, acute care hospitals for Medicare fee‐for‐service (FFS) patients aged 65 years and older hospitalized with a principal discharge diagnosis of AMI, heart failure, or pneumonia.^28^, ^29^, ^30^ The pneumonia measure cohort also includes admissions with a principal discharge diagnosis of sepsis (not including severe sepsis) that have a secondary discharge diagnosis of pneumonia coded as present on admission (POA) and no secondary diagnosis of severe sepsis coded as POA.^31^ To be included in the measure cohorts, patients must be enrolled in Medicare FFS Part A and Part B for one year before their admission date and enrolled in Part A during their index admission to ensure adequate data for risk adjustment. Patients who died during the hospitalization or were discharged against medical advice were excluded from the measure cohorts. Finally, for patients transferred to another acute care institution, we attributed the readmission outcome to the hospital that ultimately discharged the patient to a non‐acute setting.

### 2.3 Variables

The outcome of interest is 30‐day readmission following AMI, heart failure, or pneumonia hospitalization. We used the risk factors documented for each existing readmission measure, which include age, comorbidities, and prior medical history (for more details on the variables included in the risk adjustment models, see^31^). We used the “state reported dual eligible status code” variable in the MBSF to determine a patient's dual eligibility status. Dual eligible patients are defined as those patients who receive full Medicaid coverage. To identify a patient's race, we used information available in the Medicare EDB. Racial/ethnic categories available in this file include White (not Hispanic origin), Black (not Hispanic origin), Asian/Pacific Islander, Hispanic, Native American/Alaskan Native, other, and unknown. However, these data are not consistently captured in Medicare claims. Sensitivity analyses showed that it is difficult to reliably distinguish between the five aforementioned racial and ethnic groups.^32^, ^33^ White and Black patients are the only two groups with high sensitivity and specificity. Therefore, we only included patients coded as White or Black in our racial disparity analyses.

### 2.4 Underlying risk adjustment model

The method presented here is applicable to any social risk factors and dichotomous outcomes. It was developed as an extension of the risk standardized outcome measures developed and reported by CMS in measures (see readmission measures on QualityNet). These publicly reported measures include the three conditions and outcomes evaluated here: AMI, heart failure, and pneumonia readmission. Below, we describe the specific model and assumptions common to all of them.

Suppose *Y*_*ij*_ indicates whether the ith patient at the jth hospital is readmitted within 30 days, and ***Z***_***ij***_ is a vector of risk factors for that patient. Then, we would first estimate a mixed effects model:

(1) $$\underset{\gamma_{j}∼N\left(0,\tau^{2}\right)}{\text{logit}(\text{Pr}\left[Y_{\mathrm{ij}}\;=\;1\right])}\;=\;\beta_{0}\;+\;B^{T}Z_{\mathrm{ij}}\;+\;\gamma_{j}$$

where *γ*_*j*_ is a random hospital effect. The random effect *γ*_*j*_, sometimes called the “hospital‐specific effect,” can be interpreted as a latent quality trait for hospital *j* because it estimates the contribution of the hospital to the outcome risk for all patients admitted to hospital *j*. Once model (1) is estimated, it is used for these measures to calculate for each patient a predicted probability of the outcome *P*_*ij*_ and an expected probability *E*_*ij*_ where

$$P_{\mathrm{ij}}\;=\;{\text{logit}}^{-1}\left(\beta_{0}\;+\;B^{T}Z_{\mathrm{ij}}\;+\;\gamma_{j}\right);\;E_{\mathrm{ij}}\;=\;{\text{logit}}^{-1}\left(\beta_{0}\;+\;B^{T}Z_{\mathrm{ij}}\right).$$

These represent the predicted risk for patient *i* using hospital *j*'s specific latent quality and the risk predicted for the same patient assuming he or she were treated at a hospital with average latent quality. Once these are calculated, they are used to construct a standardized risk ratio (SRR) for each hospital *j*:

(2) $${\text{SRR}}_{j}\;=\;\left(\Sigma P_{\mathrm{ij}}\right)/\left(\Sigma E_{\mathrm{ij}}\right)$$

where the sum is over all patients at hospital *j*. This is usually multiplied by the overall crude rate mean (*Y*_*ij*_) to produce a risk‐standardized rate (RSRR), which is reported.

### 2.5 Disparity model

Model (1) can be expanded to include an additional risk factor *X* (e.g, dual eligibility), which captures the fixed effect of *X* on patient outcomes:

(3) $$\underset{\gamma_{j}∼N\left(0,\tau^{2}\right)}{\text{logit}(\text{Pr}\left[Y_{\mathrm{ij}}\;=\;1\right])}\;=\;\beta_{0}\;+\;{BZ}_{\mathrm{ij}}\;+\;\beta_{X}X_{\mathrm{ij}}\;+\;\gamma_{j}.$$

Here, β_*X*_ represents the *overall disparity effect*. While important to assess, it is a fixed effect, which is the same for all hospitals. To assess within‐hospital disparities related to patient attribute *X* (e.g, dual eligibility), we assume that in addition to the hospital‐specific effect described above and the fixed effect β_*X*_, there is an additional latent disparity trait at each hospital, such that patients with *X* = 1 have an increased or decreased risk of the outcome specific to that hospital:

(4) $$\underset{\left(\gamma_{j},\varepsilon_{j}\right)∼N\left(0,\Sigma^{2}\right)}{\text{logit}(\text{Pr}\left[Y_{\mathrm{ij}}\;=\;1\right])}\;=\;\beta_{0}\;+\;{BZ}_{\mathrm{ij}}\;+\;\left(\beta_{X}\;+\;\varepsilon_{j}\right)X_{\mathrm{ij}}\;+\;\gamma_{j}$$

where ε_*j*_ is the *hospital‐specific disparity effect* (or within‐hospital disparity effect) and represents the latent disparity trait for each hospital. Model (4) is known as a “mixed effects random slope model.” There are different ways of specifying the same model, but for purposes of estimation we use a form that separates the between‐hospital effect (effect of being at a hospital with a high proportion of patients with the risk factor) from the within‐hospital effect (effect of having the social risk factor at a particular hospital). In order to better interpret the results, we also center all factors ***Z***_***ij***_ on their overall mean. Thus, our final model is:

(5) $$\begin{array}{cc} \text{logit}(\text{Pr}[Y_{\mathrm{ij}}\;= & \;1])\;=\;\beta_{0}\;+\;\beta_{1}\left(Z_{ij1}-Z_{..1}\right)\;+\ldots \;+\;\beta_{p}\left(Z_{\mathrm{ijp}}-Z_{..p}\right)\;\\ & +\;\gamma_{j}\;+\;\beta_{x}\left(X_{\mathrm{ij}}-X_{j.}\right)\;+\;\beta_{x2}\left(X_{j.}-X_{..}\right)\;+\;\varepsilon_{j}\left(X_{\mathrm{ij}}-X_{j.}\right) \end{array}$$

where

- $$Z_{..k}\;=\;\frac{1}{\sum_{i=1}^{I}n_{i}}\sum_{j=1}^{I}Z_{\mathrm{ijk}}\;\text{for}\;K\;=\;1,\ldots ,p;$$
- *X*_*ij*_ is the indicator of social risk factor (e.g, 1 = dual, 0 = non‐dual or 1 = Black, 0 = White) for case i at hospital *j*;
- $X_{j.}\;=\;\frac{1}{n_{j}}\sum_{i=1}^{n_{j}}X_{\mathrm{ij}}$ is the proportion of cases with social risk factors in hospital *j* and $X_{..}\;=\;\frac{1}{I}\sum_{j=1}^{I}X_{j.}$ is the average of all hospitals proportion of cases with social risk factors;
- ($\gamma_{j},\varepsilon_{j}$)′~N_2_(0, Σ) with $\Sigma \;=\;\left(\begin{array}{cc} \sigma_{0}^{2} & \sigma_{01}\\ \sigma_{01} & \sigma_{1}^{2} \end{array}\right)$.

In this model, the fixed effect β_*x*_ reflects overall disparity, that is, the average disparity effect across all hospitals. The random slope $\varepsilon_{j}$ reflects hospital *i*'s hospital‐specific disparity effect, that is, the degree to which the disparity in outcomes in hospital *j* differs from the average disparity. By combining these two, we can estimate the disparity effect at a given hospital.

### 2.6 Reporting

Once model (5) is estimated, we propose reporting the hospital disparity using in a metric that is both accurate and accessible to consumers: the absolute rate difference (ARD). The ARD is calculated from model (5) by predicting the probability of a positive outcome under two different assumptions and calculating the difference. In both cases, we assume ***Z*** **= **mean**(** ***Z***_***ij***_ **)**, the average value of all risk factors in the population, and include the hospital‐specific quality effect *γ*_*j*_ and hospital‐specific disparity ε_*j*_. For one, we assume *X*_*ij*_ = 0 that the hypothetical patient has no disparity risk factor, and for the other, we assume *X*_*ij*_ = 1 that the hypothetical average patient has the disparity risk factor. The difference between these two predicted probabilities is the ARD, which can be intuitively interpreted as the difference in outcome rates for “average patients” treated at that hospital with and without the social risk factor. As an alternative to the ARD, we also report the hospital‐specific odds ratio, OR_*j*_ = exp(β_*x*_ + ε_*j*_), representing the odds of an average patient with the given social risk factor to be readmitted after discharge from that hospital, relative to the analogous odds for an average patient without the social risk factor. A bootstrap procedure is used to obtain the 95% confidence intervals for each hospital's odds ratio and ARD. The detailed model specifications and bootstrap procedures for identifying outlier hospitals are described in the Appendix S1.

### 2.7 Statistical analyses

For each measure described above, we summarized the number of hospitals and patients, the percent of dual eligible patients, and the observed 30‐day readmission rates. We then estimated model (5) and calculated the ARD and 95% confidence intervals for each hospital. We report the overall disparity odds ratio, variances of the hospital‐specific disparity effect, ARD distributions, and “statistical outliers” (hospitals whose 95% confidence interval for the ARD lies fully above or fully below 0). In alignment with likely public reporting thresholds, we examined the ARD distributions for hospitals with at least 25 patients overall and at least 12 patients in each subgroup. We also provide results on the relationship between overall quality and disparities. Finally, we examined correlations between socioeconomic disparities and racial disparities.

All analyses were performed using *SAS* version 9.4 (SAS Institute, Cary, NC, USA). The Human and Investigation Committee at the Yale University School of Medicine provided an exemption to use CMS claims and enrollment data for research analyses and publication.

## 3 RESULTS

### 3.1 Socioeconomic disparities

#### 3.1.1 Descriptive statistics

The study includes 501 429 admissions to 4220 hospitals for AMI, 1 162 288 admissions to 4639 hospitals for heart failure, and 1 475 989 admissions to 4692 hospitals for pneumonia. Among patients hospitalized for AMI, heart failure, and pneumonia, 14.3%, 18.7%, and 24.6%, respectively, were dual eligible. In our sample, the unadjusted readmission rates within 30 days of index discharge for all patients were 16.8%, 21.9%, and 17.1% for AMI, heart failure, and pneumonia, respectively. The unadjusted readmission rate was substantially higher among dual eligible patients compared to non‐dual eligible patients with a difference of about 5.7% for AMI, 4.0% for heart failure, and 3.1% for pneumonia (see Table 1).

**Table 1:** Within‐hospital disparities by dual eligibility for AMI, heart failure, and pneumonia readmission

	AMI	Heart failure	Pneumonia
Hospitalizations			
All	501 429	1 162 288	1 475 989
Percentage duals	14.3%	18.7%	24.6%
Observed readmission rates			
Overall	16.84%	21.93%	17.08%
Duals	21.70%	25.21%	19.38%
Non‐duals	16.03%	21.18%	16.33%
Disparity odds ratio			
Duals vs Non‐duals	1.09 (*P*‐value <0.0001)	1.07 (*P*‐value <0.0001)	1.05 (*P*‐value <0.0001)
Median hospital level absolute rate difference (Interquartile range)			
Duals vs Non‐duals	1.12% (1.09%‐1.19%)	1.10% (1.02%‐1.20%)	0.60% (0.41%‐0.83%)
Variance of the hospital‐specific disparity effect			
Dual eligibility model	0.006 (*P*‐value = 0.177)	0.004 (*P*‐value = 0.067)	0.011 (*P*‐value <0.0001)

AMI, acute myocardial infarction.

#### 3.1.2 Overall disparity effect

We began our analysis by assessing the overall disparity effect (Disparity Odds Ratio in Table 1). The overall disparity effect is fixed across hospitals and reflects health disparities by social risk factor conditional on comorbidities. The overall odds ratio between dual eligible and non‐dual eligible patients was 1.05 for pneumonia, 1.07 for heart failure, and 1.09 for AMI. Accordingly, the odds that a dual eligible patient is readmitted are 5%‐9% higher compared to non‐dual eligible patients depending on the condition, even after adjusting for comorbidities.

#### 3.1.3 The hospital‐specific disparity effect

**Figure 1:**
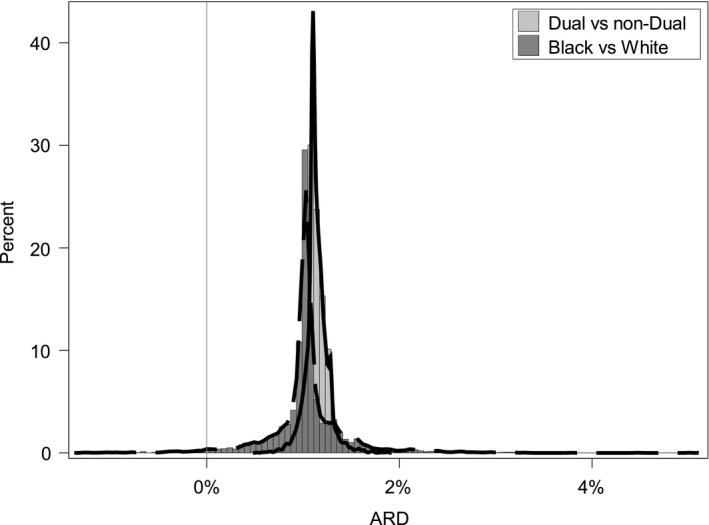
Distribution of absolute rate difference between dual and non‐dual eligible patients, and black and white patients among all hospitals for heart failure readmission (N = 4220)

**Figure 2:**
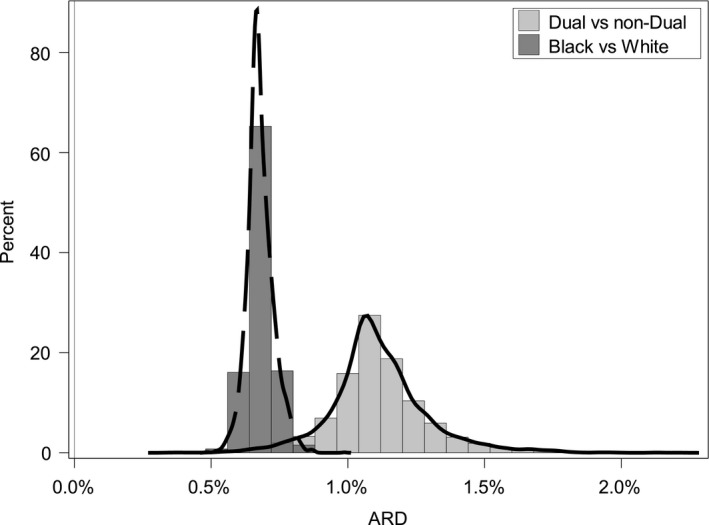
Distribution of absolute rate difference between dual and non‐dual eligible patients, and black and white patients among all hospitals for heart failure readmission (N = 4639)

**Figure 3:**
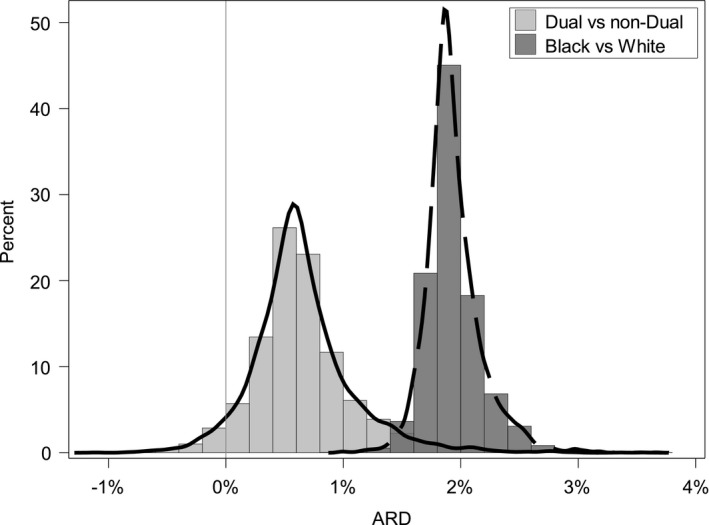
Distribution of absolute rate difference between dual and non‐dual eligible patients, and black and white patients among all hospitals for pneumonia readmission (N = 4692)

For most hospitals in our sample, the within‐hospital readmission rates were higher among dual eligible patients compared to non‐dual eligible patients across the three conditions after adjusting for comorbidities (Figures 1, 2, 3). The results show that the hospital‐specific disparity effect varied substantially across hospitals. For some hospitals, the ARD estimated from model (5) between dual and non‐dual eligible patients for pneumonia was as large as 3.6%, indicating that dual eligible patients in these hospitals are substantially more likely to be readmitted after accounting for differences in clinical factors, such as comorbidities. In other hospitals, however, the gap was as small as −0.9%. For heart failure, the hospital‐specific disparity effect measured in terms of ARD ranged from 0.33% to 2.22%, and from 0.5% to 1.9% for AMI. The variation in hospital‐specific disparities (the variance of the hospital‐specific disparity effect) was statistically significant for the pneumonia readmission measure, but not for AMI and heart failure readmission (Table 1). This means that for AMI and heart failure readmission, the effect of dual eligibility on readmission (or the hospital‐specific disparity effect) does not differ significantly across hospitals. In addition, there were a limited number of outlier hospitals across the three measures examined. The ARD and odds ratio calculations found no hospital with significant disparities for AMI readmission, one (0.02%) hospital with significant disparities in favor of non‐dual eligible patients for heart failure readmission, and seven hospitals (0.15%) with significant disparities for pneumonia readmission in favor of non‐dual eligible patients. The small numbers of outlier hospitals are likely due to the relatively small effect size of the national overall disparity across these three measures.

Table 2 reports the distribution of disparity effects for hospitals with at least 25 patients overall and 12 dual and 12 non‐dual eligible patients for the AMI, heart failure, and pneumonia readmission measures. The mean (SD), median, minimum, and maximum ARD were similar to that of the full set of hospitals.

**Table 2:** Summary of absolute rate difference in readmission between dual and non‐dual eligible patients for pneumonia, heart failure, and AMI readmission

Hospital	N	Mean	SD	Median	Min	5th Percentile	10th Percentile	25th Percentile	75th Percentile	90th Percentile	95th Percentile	Max
Pneumonia readmission												
All hospitals	4692	0.65%	0.43%	0.60%	−1.11%	0.02%	0.19%	0.41%	0.83%	1.17%	1.43%	3.56%
Hospitals reaching volume threshold	3778	0.66%	0.48%	0.61%	−1.11%	−0.03%	0.13%	0.36%	0.89%	1.24%	1.50%	3.56%
Heart failure readmission												
All Hospitals	4638	1.11%	0.18%	1.10%	0.34%	0.84%	0.92%	1.02%	1.19%	1.32%	1.43%	2.21%
Hospitals reaching volume threshold	2987	1.12%	0.21%	1.11%	0.34%	0.79%	0.87%	0.99%	1.24%	1.39%	1.50%	2.21%
Acute Myocardial Infarction (AMI) Readmission												
All hospitals	4220	1.14%	0.12%	1.12%	0.52%	0.96%	1.02%	1.09%	1.19%	1.28%	1.32%	1.88%
Hospitals reaching volume threshold	1528	1.14%	0.17%	1.13%	0.52%	0.88%	0.94%	1.03%	1.23%	1.35%	1.44%	1.88%

AMI, acute myocardial infarction.

#### 3.1.4 Relationship between with socioeconomic disparities and overall quality

The correlation between socioeconomic disparities and overall quality is positive, statistically significant, and moderate in size for heart failure (*r* = 0.47*, *P* < 0.05) and pneumonia (*r* = 0.44*, *P* < 0.05) readmission, indicating that hospitals that have high socioeconomic disparities tend to have worse hospital quality in terms of their overall RSRR. For AMI readmission, the relationship is negative and statistically significant, but the association is very weak (*r* = −0.08*, *P* < 0.05).

### 3.2 Racial disparities

#### 3.2.1 Descriptive statistics

About 8% of patients hospitalized for AMI, 13% hospitalized for heart failure, and 8% hospitalized for pneumonia were Black. The number of hospitals included in the disparity model for race was lower than the number included in the dual eligibility model because some hospitals did not provide care to any Black patients. There was a total of 4202 hospitals for the AMI cohort, 4600 hospitals for the heart failure cohort, and 4655 hospitals for the pneumonia cohort.

Across the three measures, the results indicate that Black patients were more likely to be readmitted compared to White patients (See Observed Readmission Rate in Table 2). The observed difference in readmission rates between Black and White patients was 4.8% for AMI, 2.9% for heart failure, and 4.8% for pneumonia.

#### 3.2.2 Overall disparity effect

Table 2 presents the overall disparity effect, which quantifies racial disparities within hospitals after controlling for differences in patients’ severity of illness and is fixed across hospitals (see Disparity Odds Ratio in Table 2). The overall odds ratio between Black and White patients was as high as 1.15 for pneumonia. Accordingly, the odds that a Black patient is readmitted are 15% higher compared to a White patient for this specific condition, even after adjusting for patients’ comorbidities. For heart failure and AMI, the overall odds ratio was 1.04 and 1.08, respectively, indicating that the odds that a Black patient is readmitted are 4%‐8% higher compared to a White patient.

#### 3.2.3 The hospital‐specific disparity effect

For all hospitals in our sample, within‐hospital readmission rates were higher among Black patients compared to White patients across the three conditions examined and after adjusting for patients’ comorbidities. Results indicated that the variance of the hospital‐specific disparity effect was significant for AMI and pneumonia readmission, but not for heart failure readmission (Table 3). Figures 1, 2, 3 show that hospital‐specific disparities by race varied substantially across hospitals for AMI and pneumonia readmission. The Black–White ARD in readmission ranged from 0.0% % to 5.0% for AMI. For pneumonia, the ARD varied from 1.0% to 3.7%. For heart failure, the ARD varied from 0.5% to 1.0%.

**Table 3:** Within‐hospital disparities by race for AMI, heart failure, and pneumonia readmission

	AMI	Heart failure	Pneumonia
Hospitalizations			
All	501 429	1 162 288	1 475 989
Percentage of blacks	8.3%	12.6%	7.9%
Observed readmission rates			
Overall	16.84%	21.93%	17.08%
Blacks	21.2%	24.4%	21.5%
White	16.4%	21.5%	16.7%
Disparity odds ratio			
Black vs White	1.08 (*P*‐value <0.0001)	1.04 (*P*‐value <0.0001)	1.15 (*P*‐value <0.0001)
Median hospital level absolute rate difference (Interquartile range)			
Black vs White	1.03% (0.96%‐1.12%)	0.67% (0.65%‐0.71%)	1.90% (1.80%‐2.04%)
Variance of the hospital‐specific disparity effect			
Race model	0.031 (*P*‐value <0.003)	0.001 (*P*‐value = 0.339)	0.006 (*P*‐value =0.046)

AMI, acute myocardial infarction.

We identified more outlier hospitals for the race model than for the dual eligibility model. One (0.02%) hospital had significant disparities (in favor of white patients) in AMI readmission, no hospitals had significant disparities for heart failure readmission, and 237 (5.7%) hospitals had significant disparities (in favor of white patients) for pneumonia readmission. The large number of outlier hospitals for pneumonia readmission is likely due to the relatively large effect size of the national overall disparity between Black and White patients.

Table 4 reports the distribution of disparity effects for hospitals with at least 25 patients overall and 12 Black and 12 White patients. The mean (SD), median, minimum, and maximum ARD were similar to that of the full set of hospitals.

**Table 4:** Summary of absolute rate difference in readmission between black and white patients for pneumonia, heart failure, and AMI readmission

Hospital	N	Mean	SD	Median	Min	5th Percentile	10th Percentile	25th Percentile	75th Percentile	90th Percentile	95th Percentile	Max
Pneumonia readmission												
All hospitals	4692	1.93%	0.26%	1.89%	0.92%	1.57%	1.65%	1.78%	2.04%	2.24%	2.39%	3.76%
Reporting Hospitals	1636	2.00%	0.35%	1.98%	0.92%	1.51%	1.59%	1.78%	2.19%	2.45%	2.60%	3.76%
Heart failure readmission												
All hospitals	4639	0.69%	0.10%	0.68%	0.32%	0.54%	0.58%	0.64%	0.74%	0.82%	0.88%	1.44%
Reporting hospitals	1580	0.71%	0.12%	0.70%	0.32%	0.51%	0.56%	0.63%	0.78%	0.87%	0.93%	1.18%
Acute myocardial infarction (AMI) readmission												
All hospitals	4383	0.89%	0.32%	0.89%	−0.97%	0.39%	0.58%	0.81%	0.97%	1.18%	1.37%	5.28%
Reporting hospitals	763	0.91%	0.62%	0.89%	−0.97%	−0.04%	0.17%	0.49%	1.30%	1.61%	1.94%	5.28%

AMI, acute myocardial infarction.

#### 3.2.4 Relationship between with racial disparities and overall quality

The correlation between racial disparities and overall quality is strong with a positive, statistically significant association for heart failure (0.87*, *P* < 0.05) and pneumonia (*r* = 0.67*, *P* < 0.05) readmission, indicating that hospitals that have high racial disparities tend to have worse hospital quality in terms of their overall RSRR. For AMI readmission, the relationship is negative and statistically significant, but the strength of the association is weak (*r* = −0.22*, *P* < 0.05).

### 3.3 Relationship between socioeconomic and racial disparities

Table 5 summarizes the correlation between socioeconomic and racial disparities for AMI, heart failure, and pneumonia readmission. The correlations are moderate, but positive and statistically significant for the three outcome measures examined, indicating that hospitals that have high socioeconomic disparities also have high racial disparities. The strength of the association was the weakest for AMI (*r* = 0.19) and the strongest for heart failure (*r* = 0.52). Given that there is some overlap between patients’ social risk factors, these results suggest that mechanisms driving socioeconomic and racial disparities may be partly different.

**Table 5:** Pearson correlations between socioeconomic and racial disparities (quantified by absolute rate differences) for AMI, heart failure, and pneumonia readmission

	Correlation	*P* value	95% CI
AMI	0.19	<0.0001	0.15‐0.20
Heart failure	0.52	<0.0001	0.49‐0.54
Pneumonia	0.49	<0.0001	0.47‐0.51

AMI, acute myocardial infarction.

## 4 DISCUSSION

In this paper, we proposed and evaluated a metric for quantifying within‐hospital disparities in quality outcome measures. To date, current national quality reporting efforts do not focus on evaluating health care disparities, and measurement tools have been lacking. Our approach fills this gap. In this paper, we implemented our method using two social risk factors applied to three condition‐based readmission measures. We showed that a hospital‐specific disparity metric is technically feasible, and it is effective in capturing relative differences in outcomes within hospitals for patients with and without the social risk factor. Consistent with prior research, we found that patients with social risk factors have an increased risk of 30‐day readmission relative to patients without that social risk factor in most hospitals. But, we also found sizeable variation in within‐hospital disparities across hospitals for some but not all measures: for pneumonia readmission related to dual eligibility, and for AMI and pneumonia readmission related to Black race. Variation in within‐hospital disparities demonstrates that many hospitals have an opportunity to close the gap in health outcomes among the patients they serve.

Our approach aligns with existing, publicly reported outcome measures and shares important features with these measures. For instance, we account for differences in patients’ prior medical history to isolate the hospital‐specific disparity effect related to social risk factors. We also use mixed effects models, but extend these models using additional parameters that allow us to separate the disparity effect into a within‐hospital effect and a between‐hospital effect. In addition, by separating both from the overall hospital quality effect, we have reduced the risk of bias that might be introduced if the within‐hospital disparity was influenced by overall quality. This approach can be adapted to assess disparities for other outcome measures (such as mortality or complication measures) and for any social risk factor (e.g, spoken language or gender). It provides a framework for assessing within‐hospital disparities across different health outcomes and social risk factors. As demonstrated in our findings, we expect that variation in the hospital‐specific disparity effect will be specific to the outcome measure. At the same time, the demonstrated correlation across social risk factors for all three conditions validates our approach. Finally, the correlation between within‐hospital disparities and overall quality varies in strength across social risk factors and across the three measures. In most cases, disparities are higher in hospitals with poor overall performance. However, there are hospitals that have good overall performance and medium or high disparities, or hospitals that have poor overall performance and no within‐hospital disparities. Accordingly, disparity measures provide additional and supplementary information on hospital performance.

We also proposed and illustrated two different reporting options, namely ARD and odds ratio. The ARD is estimated directly from the model parameters and has the advantage of reflecting a quantity that is easily understood by consumers. However, especially for purposes of additional research, other reporting methods, such as odds ratio, may be more practical. We examined statistical outliers as a means of identifying hospitals with statistically significant within‐hospital disparities using a conservative method. In the context of reporting such information nationally, a number of options are feasible depending on policy goals. In addition to using lower threshold confidence intervals (e.g, 90% confidence intervals) which could identify more outliers, it may also be useful to assess performance directly using the ARD or to assess disparity performance in combination with overall performance in order to account for ceiling or floor effects (hospitals which do very poorly overall may have little within‐hospital disparity because of little within‐hospital variation). Pooling the disparity metric, either by conditions, outcomes, or social risk factors, might also provide a better picture of how well a hospital is able to mitigate risks associated with these factors.

### 4.1 Implications

Mandated by the Improving Medicare Post‐Acute Care Transformation Act (IMPACT Act) of 2014 (H.R. 4994), the Assistant Secretary for Planning and Evaluation recently recommended, among other initiatives, introducing health equity measures to illuminate disparities in health care quality. In response to these recommendations, several national initiatives aim to account for the effect of social risk factors on Medicare quality and payment programs. Our measurement approach is aligned with these recommendations. It sets the foundation to target quality improvement efforts to reduce health disparities. Paired with overall quality measures, it can be used by hospitals and policy makers to reduce gaps in health care quality and outcomes among patients.

Reporting within‐hospital disparities gives hospitals an important and actionable metric. It provides information to support and incentivize improving equity in outcomes among their own patients. However, we note that these metrics are ideally reported in tandem with related measures, both overall quality metrics and between‐hospital disparity measures. Reporting in conjunction with measurements of overall hospital quality will ensure that all groups of patients receive high‐quality care. Reporting in conjunction with measures that capture comparative quality for patients with social risk across institutions (“between‐hospital” disparities) will provide consumers and policy makers with a better understanding of both equity and expected outcomes for patient subgroups.

### 4.2 Limitations

Our proposed approach to measure within‐hospital disparities has several limitations. First, our approach is limited by the availability of information on social risk factors in claims data. However, the two social risk factors used in this article, dual eligibility and race, are generally available and accurately measured in claims data.^34^ These two factors capture patient attributes for which there is strong evidence of substantial disparities in health outcomes.^11^, ^12^, ^13^, ^14^, ^15^ Related to this is the potential *heterogeneity* of effect of social risk factors. For example, dual eligibility status may carry less information in some states, as the threshold for qualifying for Medicaid may be lower. In addition, race may represent different social risk in different regions of the United States because of relative differences in race‐related income inequality. However, this is a limitation to studying national disparities using any social risk factor.

Second, the number of patients required to construct a metric that is meaningfully precise is an important limitation. Similar to sample size limitations for other outcome measures, measuring disparities for hospitals with a small volume of patients with social risk factors is challenging. In this article, we did not impose any threshold for sample size, but publicly reported disparity measures should require a carefully selected threshold. In our sensitivity analyses, we examined results for hospitals with at least 25 patients overall and 12 patients in each subgroup. For all three outcome measures, socioeconomic and racial disparity results were similar among all hospitals and hospitals meeting the threshold requirement.

Third, the measured disparities represent observed differences in outcomes between different patient groups after accounting for multiple factors, including hospitals’ proportion of patients with social risk factors and patients’ comorbidity burden, to isolate the area under hospitals’ influence. However, a confounder may exist which influences both the risk factor and hospital readmission. Related to this is the limitation of the outcome, 30‐day readmission, which does not fully reflect the competing risk of mortality. However, as with the currently publicly reported measures, it would be meaningful to report mortality disparities based on the same method as a complimentary metric.

Another practical limitation is that we did not account for co‐occurrence of social risk factors. Some patients were both dual eligible and African American and, thus, either effect may have reflected in part that of the other risk factor. More importantly, both sets of patients may share some third social risk factor, which drives both effects. However, for purposes of promoting health equity and consumer transparency, we argue that reporting results for multiple social risk factors, as we have done, is the best way to address this limitation. Specifically, reporting separate results for each subgroup of patients makes it easier for hospitals to isolate the effect of socioeconomic and racial disparities on readmission. Disparity results are also easier to interpret by consumers when reported separately across multiple social risk factors. Thus, despite these limitations, the proposed measure provides the strongest available signal for health disparities.

## 5 CONCLUSION

Using a novel method to isolate and estimate within‐hospital disparities, we found that for 30‐day readmission these disparities vary across hospitals for some conditions and social risk factors. This method thus has the potential to incentivize the reduction of health care disparities through public reporting.
