# Supplementary material for: Measuring hospital‐specific disparities by dual eligibility and race to reduce health inequities
Source: Health Serv Res. 2019 Jan 21;54(Suppl 1):243–54. doi: 10.1111/1475-6773.13108 (PMC6341208; doi:10.1111/1475-6773.13108)
Supplement: Supplementary file 2 [file HESR-54-243-s002.docx]

**APPENDIX**

**Details for the bootstrap procedure for identification of outlier hospitals**

Based on model (5) in the Disparity Model section, for hospital j, the absolute rate difference (ARD) is calculated as

$${ARD}_{j}=\mathrm{logit}^{-1}\left[ \beta_{0}+\gamma_{j}+\beta_{x}\left( 1-X_{j\cdot} \right)+\beta_{x2}\left( X_{j\cdot}-X_{..} \right)+\epsilon_{j}\left( 1-X_{j\cdot} \right) \right]$$

$-\mathrm{logit}^{-1}\left[ \beta_{0}+\gamma_{j}-\beta_{x}X_{j\cdot}+\beta_{x2}\left( X_{j\cdot}-X_{..} \right)-\epsilon_{j}X_{j\cdot} \right]$.

The odds ratio is calculated as

$$\mathrm{OR}_{i}=\exp\left( \beta_{x}+\epsilon_{j} \right).$$

The confidence interval for both ARD and odds ratio for each hospital are calculated by the following bootstrap procedure.

Steps 1 – 4 below are repeated for b = 1, 2, …, B.

1. Sample *I* hospitals with replacement to create the *b-*th bootstrap sample.
2. Fit model (5) to the *b-*th bootstrap sample. Treat the same hospital sampled multiple times in Step 1 as distinct hospitals so that there are still *I* pairs of random effects $\left\{ \left( \gamma_{j}^{(b)},\epsilon_{j}^{(b)} \right);j=1,2,\ldots,I \right\}.$Step 2 yields:
3. The estimated fixed effects, ${\hat{\boldsymbol{\beta}}}^{(b)}=\left( \hat{\beta}_{0}^{(b)},\ldots,\hat{\beta}_{p}^{(b)} \right)^{T}$, $\hat{\beta}_{x}^{(b)}$, and $\hat{\beta}_{x2}^{(b)}$.
4. The estimated covariance matrix of random effects $\hat{\Sigma}_{11}^{(b)}$.
5. The estimated random effects and associated prediction variance of random effects, $\left\{ \hat{\gamma}_{j}^{(b)}, \hat{var}\left( \hat{\gamma}_{j}^{(b)}-\gamma_{j}^{(b)} \right); \epsilon_{j}^{(b)}, \hat{var}\left( \hat{\varepsilon}_{j}^{(b)}-\epsilon_{j}^{(b)} \right);j=1,2,\ldots,I \right\}$
6. For the unique set of hospitals sampled in Step 1, generate a new random intercept estimate $\hat{\gamma}_{j}^{(b*)}$ as a single random draw from N$\left( \hat{\gamma}_{j}^{(b)}, \hat{var}\left( \hat{\gamma}_{j}^{(b)}-\gamma_{j}^{(b)} \right) \right)$ and *independently* generate a new random slope estimate $\hat{\epsilon}_{j}^{\left( b* \right)}$ as a single random draw from N$\left( \hat{\epsilon}_{j}^{\left( b \right)}, \hat{var}\left( \hat{\varepsilon}_{j}^{(b)}-\epsilon_{j}^{(b)} \right) \right)$.
7. For the unique set of hospitals sampled in Step 1, calculate the b-th bootstrap replicate:

${OR}_{j}^{\left( b \right)}=exp(\hat{\beta}_{x}^{(b)}+\hat{\epsilon}_{j}^{\left( b* \right)})$,

$${ARD}_{j}^{\left( b \right)}= \mathrm{logit}^{-1}\left[ \hat{\beta}_{0}^{(b)}+\hat{\gamma}_{j}^{(b*)}+\hat{\beta}_{x}^{(b)}\left( 1-X_{j\cdot} \right)+\hat{\beta}_{x2}^{(b)}\left( X_{j\cdot}-X_{..} \right)+\hat{\epsilon}_{j}^{\left( b* \right)}\left( 1-X_{j\cdot} \right) \right]$$

$-\mathrm{logit}^{-1}\left[ \hat{\beta}_{0}^{(b)}+\hat{\gamma}_{j}^{(b*)}-\hat{\gamma}_{10}^{(b)}X_{j\cdot}+\hat{\beta}_{x2}^{(b)}\left( X_{j\cdot}-X_{..} \right)-\hat{\epsilon}_{j}^{\left( b* \right)}X_{j\cdot} \right]$.

After Steps 1-4 are repeated for B times, different hospitals get different numbers of OR and ARD replicates. Let $B_{min}$ denote the smallest number of OR and ARD replicates ($B_{min}$ is typically slightly above 2400 for B=4000). For each hospital, we randomly select $B_{min}$ OR and ARD replicates from all its available OR and ARD replicates, so that all hospitals use $B_{min}$ OR and ARD replicates to construct CIs. The 95% CI of OR (ARD) for a hospital is constructed as interval between the 2.5 percentile and 97.5 percentile of that hospital’s $B_{min}$ OR (ARD) replicates.
